# Polymeric Nanovehicle of α-Tocopheryl Succinate Based on a Methacrylic Derivative of Hydroxychloroquine and Its Cytotoxic Effect on Breast Cancer Cells

**DOI:** 10.3390/polym17192672

**Published:** 2025-10-02

**Authors:** Hernán Valle, Raquel Palao-Suay, Jesús Miranda, María Rosa Aguilar, Manuel Palencia

**Affiliations:** 1 Chemistry Department, Faculty of Basic Sciences, University of Córdoba, Montería 230002, Colombia; jesusandres2002.jm@gmail.com; 2 Instituto de Ciencia y Tecnología de Polímeros (ICTP), CSIC, c/Juan de la Cierva 3, 28006 Madrid, Spain; rpsuay@ictp.csic.es (R.P.-S.); mraguilar@ictp.csic.es (M.R.A.); 3 Centro de Investigación Biomédica en Red de Bioingeniería, Biomateriales y Nanomedicina (CIBER-BBN), Instituto de Salud Carlos III, 28029 Madrid, Spain; 4 Research Group in Science with Technological Applications (GI-CAT), Department of Chemistry, Faculty of Natural and Exact Sciences, Universidad del Valle, Cali 25360, Colombia; manuel.palencia@correounivalle.edu.co

**Keywords:** biomedical applications, copolymers, nanoparticles, radical polymerization, self-assembly, anticancer

## Abstract

This study focuses on the preparation of poly(HCQM-*co*-VP) copolymeric nanoparticles (NPs) to enhance the aqueous solubility and bioavailability of the hydrophobic and antitumor molecules HCQ (hydroxychloroquine) and α-TOS (α-tocopheryl succinate). HCQ is covalently incorporated into the polymer backbone, while α-TOS is encapsulated within the nanoparticles by non-covalent interactions. Poly(HCQM-*co*-VP) was synthesized from a vinyl derivative of HCQ (HCQM) and N-vinylpyrrolidone (VP), with a molar composition of 17% HCQM and 83% VP, providing the optimal hydrophobic/hydrophilic balance for forming, via nanoprecipitation, empty nanoparticles (NPs) with a diameter of 123.6 nm and a zeta potential of −5.8 mV. These nanoparticles effectively encapsulated α-TOS within their hydrophobic core, achieving an encapsulation efficiency (%EE) of 78%. These α-TOS-loaded NPs resulted in smaller diameters and more negative zeta potentials (71 nm, −19.2 mV) compared to the non-loaded NPs. The cytotoxicity of these NPs was evaluated using the AlamarBlue assay on MCF-7 breast cancer cells. The empty NPs showed no toxic effects within the tested concentration range, after 72 h of treatment. In contrast, the α-TOS-loaded NPs, exhibited a pronounced cytotoxic effect on MCF-7 cells with an IC_50_ value of 100.2 μg·mL^−1^, thereby demonstrating their potential as controlled drug delivery systems for cancer treatment. These findings contribute to the development of a new HCQ-based polymeric nanocarrier for α-TOS or other hydrophobic drugs for the treatment of cancer and other diseases treatable with these drugs.

## 1. Introduction

Cancer is the second leading cause of death worldwide, with 9.74 million deaths in 2022, a figure projected to rise to 16.9 million by 2045 [1]. This disease is characterized by the rapid proliferation of abnormal cells and their spread through metastasis. The most common types of cancer include breast, lung, colorectal, prostate, skin, and gastric cancers [2]. Despite advancements in cancer treatment, conventional chemotherapy continues to face significant challenges, such as frequent cellular drug resistance, high cytotoxicity, and low selectivity toward cancerous cells. These issues lead to adverse effects in patients, including neurotoxicity, cardiotoxicity, and nephrotoxicity [3,4].

The high cytotoxicity of conventional anticancer agents is primarily attributed to their hydrophobic nature and low molecular weight (<500 Da) (e.g., paclitaxel, doxorubicin, cisplatin, etc.). To address drug resistance, combination chemotherapy is increasingly employed, leveraging the simultaneous administration of two or more free drugs with different mechanisms of action to enhance therapeutic effects compared to monotherapy, which uses a single drug.

Nanomedicine has emerged as a revolutionary tool for cancer treatment, enabling the targeted delivery of drugs through nanoparticles (NPs) specifically directed to tumors via the enhanced permeability and retention (EPR) effect [5]. Nanoparticles with diameters ranging from 50 to 150 nm are considered optimal for EPR-mediated tumor targeting [6]. This effect arises from unique characteristics of cancerous tissue, such as increased vascular permeability, the acidic pH of the tumor microenvironment (TME), abnormal vasculature architecture, and impaired lymphatic drainage, which facilitate the efficient accumulation of NPs and controlled drug release at the tumor site. In contrast, healthy tissues’ structural integrity prevents drug accumulation, causing smaller drug molecules to disperse more freely, reducing selectivity, and accelerating elimination from the body [3].

Nanoparticulate materials can be categorized based on their chemical nature into inorganic and organic types. Among organic nanoparticles, polymeric nanoparticles have gained significant attention in recent years due to their utility as vehicles for delivering antitumor drugs. Drug incorporation into polymeric nanoparticles can occur via covalent bonding (“polymeric prodrug nanoparticles” (PDNP)) or non-covalent intermolecular forces [5,7]. Additionally, PDNPs that physically encapsulate a second anticancer drug could produce a potential synergistic therapeutic effect by combining the actions of both drugs (the covalently linked and the physically encapsulated). This combined effect would contribute to enhancing antineoplastic efficacy and minimizing the resistance to (free) drugs that these types of cells typically exhibit [5,8].

In recent years, there has been a significant increase in research focused on developing anticancer polymeric nanoparticles derived from bioactive quinoline molecules [9,10,11,12]. A notable example of such molecules is hydroxychloroquine (HCQ), an antimalarial drug that gained global attention during the COVID-19 pandemic as a potential treatment against the virus. HCQ is also an anticancer agent whose mechanism of action involves autophagy inhibition and lysosomal pH disruption [13]. Nevertheless, this molecule is known for its cytotoxicity (low selectivity toward cancer cells) and poor aqueous solubility, making it a compelling candidate for incorporation into a polymeric prodrug nanoparticle (PDNP) system to address its limitations as a free molecule.

To date, there is little information published about anticancer PDNPs based on HCQ, including those based on both synthetic and natural polymers. Wang et al. developed PDNPs using an amphiphilic polymer obtained via RAFT polymerization between a polyethylene glycol-derived chain transfer agent and vinyl monomer derived from HCQ containing a redox-sensitive disulfide spacer (also sometimes called a linker) in its structure. Using the same strategy, the anticancer drug SN38 (a camptothecin derivative) was incorporated into a second polymer, which was then co-assembled with the HCQ-derived polymer, generating a combined prodrug nanoparticle system that exhibited a synergistic cytotoxic effect against triple-negative breast cancer cells [14].

Xie et al. synthesized the polycation PDC17-H by RAFT copolymerization of the vinyl monomers DMAEMA and HCQ methacrylate, which was subsequently combined with the therapeutic microRNA anti-miR-210 (polyanion) to generate a nanoscale anticancer complex (polyplex) with dual functionality. PDC17-H enhanced the uptake and cytoplasmic release of anti-miR-210 in MDA-MB-231 cancer cell, thereby blocking the pro-tumoral activity of its therapeutic target miR-210, which is hypoxia-inducible. Moreover, it inhibited cancer cell migration even when administered alone as a PDNP, without microRNA loading. This antimetastatic effect was attributed to the HCQ moiety conjugated within the copolymer [15].

The molecular structure of both previously described PDNPs comprised synthetic polymers produced by RAFT (reversible addition-fragmentation chain transfer polymerization) polymerization. However, despite providing good control over the molecular weight and polydispersity of the resulting polymers, the RAFT technique has certain drawbacks, such as high cost and its limited applicability to a specific set of monomers [16].

Other anticancer PDNPs based on HCQ have been developed by covalently conjugating HCQ to biopolymers such as starch and hyaluronic acid [17,18]. The main drawback of these natural polymer-based nanosystems lies in the frequent batch-to-batch variability of their properties (e.g., nanoparticle size, drug loading, and release rates), which hinder their standardized application in nanomedicine [19,20].

In this work, PDNPs based on HCQ were obtained for the first time via conventional free radical polymerization (CFRP) between VP (N-vinylpyrrolidone) and a methacrylic derivative of HCQ, previously synthesized by esterification of HCQ with 2-(methacryloyloxy)ethyl monosuccinate carboxylic acid (MES). Notably, this monomer introduces a succinyl spacer susceptible to hydrolysis, enabling HCQ release. The combination of CFRP, the succinyl spacer, and the selected comonomer, represents the novelty of the present study with respect to the systems described previously. In contrast to RAFT polymerization, CFRP offers lower cost, greater simplicity, and the ability to polymerize a broader range of vinyl monomers.

In addition, the amphiphilic copolymer poly(HCQM-*co*-VP) was evaluated for its potential ability to self-assemble into nanometer-sized micelles in aqueous medium, reduce hydrophobicity, and enhance the anticancer activity of free HCQ through the EPR effect. The hydrolysable spacer incorporated in the present PDNP design is intended to enable controlled HCQ release within the tumor microenvironment via acid- or enzyme-mediated ester hydrolysis. Furthermore, the selection of VP as a comonomer is based on its well-known propensity to form stable and biocompatible nanoparticles in aqueous systems.

Another novel aspect of the present study is that it also evaluates the ability of poly(HCQM-*co*-VP) to encapsulate the anticancer drug α-TOS and reduce the viability of MCF-7 cells (breast adenocarcinoma) through the in vitro AlamarBlue assay. α-TOS is a vitamin E analog that has shown the ability to induce apoptosis via complex II of the mitochondrial electron transport chain, and suppress cancer cell proliferation in humans in a highly selective manner, with little to no effect on normal cells. This drug is more cost-effective to produce compared to conventional antineoplastic agents due to facile semisynthesis from vitamin E. However, a significant limitation of α-TOS is its low solubility in aqueous media, which reduces its bioavailability [21]. Nanoencapsulation thus provides an effective and stable method for its administration.

**Figure 1:**
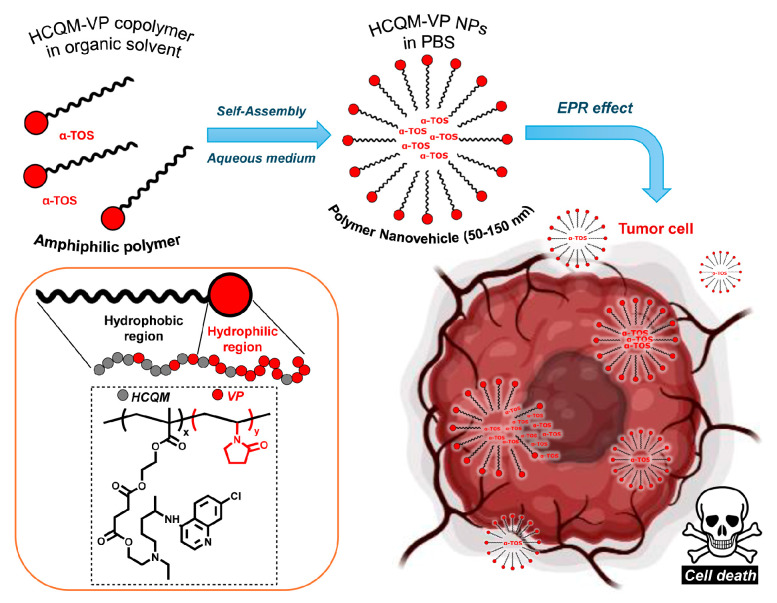
Schematic representation of the self-assembly by nanoprecipitation of an amphiphilic copolymer formed by a derivative of HCQ and VP, capable of encapsulating α-TOS and reducing tumor cell viability.

Although a formal synergy between HCQ and α-TOS has not been reported, their distinct mechanisms of action (autophagy inhibition and mitochondrial apoptosis, respectively) may act in a complementary manner. Indeed, it has been reported that autophagy inhibition can sensitive tumor cells to apoptotic cell death [22]. A schematic summary of this work is shown in Figure 1.

## 2. Experimental

### 2.1. Materials and Reagents

For the synthesis of the HCQM monomer, a variety of reagents and solvents were used, which were directly obtained from commercial suppliers without undergoing any additional purification processes. These include hydroxychloroquine sulfate (HCQ) supplied by Acros Organics (Fair Lawn, NJ, USA) and 1-ethyl-3-(3′-dimethylaminopropyl)-carbodiimide (EDC), 4-dimethylaminopyridine (DMAP), Mono-(2-(methacryloyloxy)ethyl)succinate (MES), tetrahydrofuran (THF), anhydrous N,N-dimethylformamide (DMF), dichloromethane (DCM), supplied by Sigma (Burlington, MA, USA and St. Louis, MO, USA), as well as n-hexane, anhydrous sodium sulfate (Na_2_SO_4_), and sodium bicarbonate (NaHCO_3_), which were produced by Merck (Darmstadt, Germany). The thin-layer chromatography (TLC) technique was applied to confirm the identity and purity of the compounds using aluminum-backed silica gel Merck 60 F254 plates of 10 by 5 cm (Merck, Darmstadt, Germany) and a 254/366 nm dual-wavelength UV lamp (Camag, Muttenz, Switzerland) as the visualizing agent for the chromatograms. For the synthesis and purification of HCQM-VP copolymers and homopolymers, N-vinylpyrrolidone (VP), DMSO, and 1,4-dioxane, manufactured by Merck (Darmstadt, Germany), were used and purified by vacuum distillation. Additionally, the initiator 2,2′-azobisisobutyronitrile (AIBN, Merck, Darmstadt, Germany) was recrystallized in methanol. Dialysis membranes from Spectra/Por with a molecular weight cutoff of 3.5 kDa MWCO (Spectruab, Rancho Dominguez, CA, USA) were also utilized.

### 2.2. Spectroscopic Characterization

All ^1^H NMR (proton nuclear magnetic resonance) analyses were recorded at 25 °C using a 400 MHz Bruker Ascend™ NMR spectrometer (Bruker Corporation, Karlsruhe, Germany). Deuterated chloroform (CDCl_3_, 98%; Sigma-Aldrich, St. Louis, MO, USA) or deuterated dimethyl sulfoxide (DMSO-*d*_6_, MagniSolve, 99.96%, Zurich, Switzerland) was used as the solvent. The samples were prepared at a concentration of 30 mg mL^−1^ in the corresponding deuterated solvent. Chemical shifts were expressed as δ values in parts per million (ppm) relative to residual solvent peaks (CDCl_3_ o DMSO-*d*_6_), and coupling constants (J) were reported in Hertz (Hz). The following abbreviations were used to describe the ^1^H NMR spectra: (s) singlet, (d) doublet, (dd) double doublet and (m) multiplet. The MestReNova software (version 6.0.2, Mestrelab Research, Santiago de Compostela, Spain) was used to normalize and integrate the spectral signals. Additionally, the quantification of the comonomer composition in the various copolymers was performed by analyzing the normalized signal areas corresponding to each comonomer present in the respective spectrum.

### 2.3. Synthesis of HCQM

**Scheme 1:**
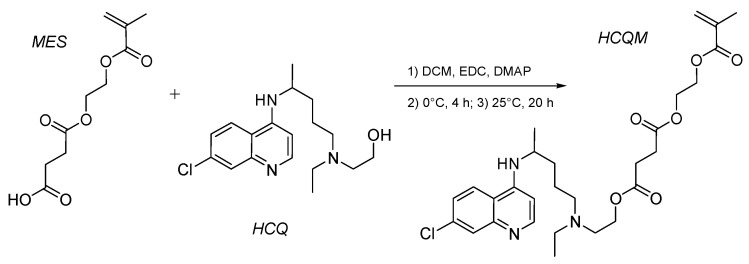
Synthesis of the HCQM monomer.

The conversion of HCQ sulfate to HCQ was performed following the methodology described by Che et al. [23]. To synthesize the HCQM monomer, an esterification reaction was carried out between mono-(2-(methacryloyloxy)ethyl) succinate (MES) and HCQ, using a modified version of the method reported by Palao-Suay et al. [24]. The chemical reaction is depicted in Scheme 1.

Initially, 7.53 g of HCQ (1.0 eq), 6.07 mL of MES (1.4 eq), 0.822 g of DMAP (0.3 eq), and 360 mL of DCM were mixed in a 1000 mL three-neck round-bottom flask, previously oven-dried and equipped with a magnetic stirring bar. After sealing the flask with a septum and purging it with an inert nitrogen atmosphere, the reagents were dissolved under stirring at 0 °C. Subsequently, 100 mL of an EDC solution (1.5 eq, 6.44 g) in dichloromethane was added via syringe through the septum under continuous stirring. After 4 h of stirring, the cooling was discontinued, and the reaction mixture was stirred for an additional 20 h at room temperature.

Subsequently, the resulting mixture was transferred to a 2000 mL separatory funnel and subjected to a series of washes: first with a saturated NaHCO_3_ solution (3 × 500 mL), followed by distilled water (2 × 250 mL), and finally with brine (2 × 500 mL). The organic phase obtained (900 mL) was dried over anhydrous sodium sulfate for 24 h. The solution was then filtered using Whatman No. 2 filter paper, and 40 mg of hydroquinone was added to prevent autopolymerization. The filtrate was concentrated under reduced pressure using a rotary evaporator at 40 °C, and the resulting product was vacuum-dried in a desiccator over phosphorus pentoxide, yielding the desired compound, HCQM.

### 2.4. Synthesis of Copolymers of HCQM and VP

**Scheme 2:**
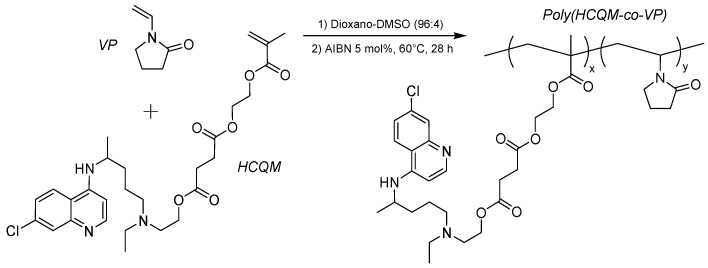
Synthesis of the poly(HCQM-*co*-VP) copolymers.

The synthesis of the poly(HCQM-*co*-VP) copolymers was performed through conventional radical polymerization (CFRP) of the HCQM and VP monomers (see Scheme 2) using 1,4-dioxane-DMSO (96:4) as the solvent. The copolymers were prepared with the following comonomer feed compositions: 5:95, 10:90, 20:80, 30:70, 50:50, 70:30, and 80:20 mol% [HCQM]:[VP]. Additionally, the homopolymers PVP and HCQM were synthesized. The total monomer concentration in the feed was 0.2 M, and AIBN (2,2′-azobisisobutyronitrile) was used as the radical initiator at 5 mol% relative to the total monomer concentration. The polymerization reactions were conducted at 60 °C for a duration of 28 h to ensure high monomer conversion. After the reaction, the products were subjected to dialysis against water for 48 h using a 3.5 kDa molecular weight cutoff (MWCO) membrane, with water being replaced every 2 h. Upon completion, the dialyzed material was lyophilized, yielding the dry copolymer.

### 2.5. Gel Permeation Chromatography (GPC)

The apparent average molecular weight (M_n_ and M_w_) and the polydispersity index (Ð) of the HCQM-derived copolymer were determined using a gel permeation chromatography (GPC) system. The analysis was performed with an HPLC instrument (PerkinElmer model 200, Waltham, MA, USA) comprising several components: a 200-series refractive index detector, an LC-250 isocratic pump, and a set of three polystyrene-divinylbenzene columns (Styragel^®^ HR, Waters Corporation, Milford, MA, USA) serving as the stationary phase.

The mobile phase used was dimethylformamide (DMF, Scharlau, Barcelona, Spain), which was pre-degassed and supplemented with 0.1% *w*/*v* LiBr (Sigma, St. Louis, MO, USA). The system operated at a constant flow rate of 0.7 mL min^−1^ and was maintained at 70 °C. System calibration was performed using monodisperse polystyrene standards (Agilent Technologies, Santa Clara, CA, USA) with molecular weights ranging from 2.93 to 3039 kDa to generate the corresponding calibration curve. Finally, the experimental data were processed and analyzed using the TotalChrom Workstation version 6.3.1 software (PerkinElmer Inc., Shelton, CT, USA).

### 2.6. Differential Scanning Calorimetry

The glass transition temperature (T_g_) of each polymer was determined using differential scanning calorimetry (DSC) with a Discovery DSC25 system (TA Instruments, New Castle, DE, USA). Approximately 5–10 mg of each sample was weighed, sealed in a Tzero aluminum pan, and heated at a constant rate of 20 °C min^−1^ from −20 to 190 °C. The resulting data were processed using TRIOS software (version 4.0, TA Instruments, New Castle, DE, USA). The T_g_ was identified as the midpoint of the heat capacity transition.

### 2.7. Thermogravimetric Analysis (TGA)

Thermogravimetric analysis was performed to evaluate the thermal stability of the polymers. Approximately 5 mg of each sample were heated from 25 to 600 °C at a rate of 10 °C min^−1^ under a nitrogen atmosphere (50 mL min^−1^) using a TGA Q500 instrument (TA Instruments, New Castle, DE, USA). Data regarding the onset of thermal decomposition and mass loss were obtained and processed using TA Instruments Universal Analysis software, version 4.5A.

### 2.8. Preparation and Characterization of Copolymer Nanoparticles

The nanoparticles (NPs) were prepared from the copolymers dissolved in THF:DMF (7:3) or THF:DMSO:DMF (86:7:7). The final concentration of the copolymer in this organic phase was 5 mg mL^−1^. Subsequently, 2.5 mL of each organic solution was added (drop by drop with constant stirring) to 10 mL of phosphate-buffered saline (PBS) solution at pH 7.4, maintaining a volume ratio of 1:4 between the organic phase and PBS. The NP suspensions were dialyzed against PBS using a 3.5 kDa (MWCO) dialysis membrane for 24 h to remove organic solvents. Nanoparticles (NPs) with a size smaller than 150 nm and no precipitate after 3 days of preparation were selected to encapsulate the anticancer drug α-TOS. The average diameter, size distribution, polydispersity index, and zeta potential of these NPs were measured using a Nanosizer NanoZS instrument (Malvern Instruments, Malvern, UK). The instrument operates based on dynamic light scattering (DLS) and electrophoretic mobility techniques. In addition, morphological characterization of the NPs (at 0.01 mg mL^−1^) was carried out by scanning electron microscopy (SEM) using a Hitachi SU8000 TED cold-emission field emission SEM microscope (Hitachi, Tokyo, Japan) working at an accelerating voltage 3.0 kV.

To encapsulate α-TOS (Sigma-Aldrich, St. Louis, MO, USA), a 10% mass ratio (relative to the polymer) of this drug was dissolved together with the copolymer in THF:DMF (7:3), and 2.5 mL of this organic solution was added drop by drop with constant stirring to 10 mL of PBS pH 7.4. The remaining steps outlined in the previous paragraph, including the removal of organic solvent and non-encapsulated α-TOS by dialysis, were then followed to obtain the drug-loaded NPs [12].

### 2.9. Determination of Encapsulation Efficiency (%EE) of α-TOS

The amorphous powder obtained after lyophilizing the α-TOS-loaded NPs was dispersed in ethanol under magnetic stirring for 24 h. After centrifugation (8000 rpm), the supernatant was analyzed using a NanoDrop™ Onec Microvolume UV-Vis Spectrophotometer (Thermo Scientific™, Waltham, MA, USA) to determine the α-TOS concentration (λ_max_ = 285 nm).

The latter was performed by replacing the absorbance value of the sample into the equation of a calibration curve based on serial ethanol dilutions of an α-TOS standard between 1 and 0.001 mg mL^−1^ (extinction coefficient = 2.050). The encapsulation efficiency (%*EE*) of α-TOS was calculated by dividing the concentration of α-TOS obtained from the calibration curve equation ([*D*]*_exp_*, mg mL^−1^) by the initial concentration of α-TOS set during nanoprecipitation ([*D*]_0_, mg mL^−1^), according to the following equation:

(1) $$\%\;EE\;=\frac{{\left[\;D\;\right]}_{exp}}{{\left[\;D\;\right]}_{0}}\;\times \;100\%$$

The final value reported will correspond to the average of three analytical replicates carried out under the same experimental conditions.

### 2.10. Cell Culture and Biological Reagents

The biological reagents and culture media were sourced from various suppliers. Dulbecco’s Modified Eagle Medium (DMEM), penicillin/streptomycin (P/S), L-glutamine, fetal bovine serum (FBS), and heparin were provided by Sigma-Aldrich (St. Louis, MO, USA). Glutamax I was obtained from Life Technologies (Waltham, MA, USA). Additionally, phenol red-free DMEM was supplied by Gibco-Thermo Fisher Scientific (Lafayette, CO, USA). Trypsin-EDTA solution was also purchased from Sigma-Aldrich (St. Louis, MO, USA), and AlamarBlue^®^ was acquired from Invitrogen (Eugene, OR, USA).

### 2.11. Cytotoxicity Assay on MCF-7 Cells

The potential cytotoxic effect of nanoparticles and the drugs α-TOS and HCQ was evaluated on MCF-7 cancer cell line. MCF-7 cells were derived from human mammary adenocarcinoma and were supplied by the European Collection of Cell Cultures (ECACC). The cells were maintained at 37 °C in a humidified atmosphere with 5% CO_2_. The Dulbecco’s modified Eagle’s medium (DMEM), enriched with 10% fetal bovine serum (FBS), 2% L-glutamine, and 1% penicillin/streptomycin (P/S), was used to culture MCF-7 cells.

The NP suspensions were filtered using 0.22 µm polyethersulfone syringe filters (Millex-GP PES Millipore Express, Merck, Darmstadt, Germany) and subsequently diluted in PBS to obtain concentrations of 500, 250, 125, 63, and 31 μg mL^−1^. Regarding HCQ, concentrations in culture medium of 672, 336, 168, 84, 42, 21, 10.5, 5.3, 2.6, 1.3, 0.7 μg mL^−1^ were evaluated. Culture medium was used to dissolve HCQ. Regarding α-TOS, concentrations ranging 53 to 0.05 μg mL^−1^, prepared in 1% DMSO medium by 2-fold serial dilution from a 53 μg mL^−1^ starting stock solution, were evaluated.

Prior to this, 6000 MCF-7 cells were seeded into each well of a 96-well culture plate. After 24 h of incubation, the culture medium in each well was replaced with a mixture of fresh medium and NPs in a 1:1 (*v*/*v*) ratio, or with the previous dilutions of the free drug (HCQ or α-TOS), as appropriate. At 72 h, the medium with the treatment was removed, the cells were washed with PBS, and a 10% AlamarBlue^®^ solution in phenol red-free DMEM was added. Following an additional 3 h of incubation, the plates were analyzed using a fluorometer at an excitation/emission wavelength of 530/590 nm (Biotek Synergy HT, BioTek Instruments, Winooski, VT, USA). For empty NP, α-TOS-loaded NP, and free drug (α-TOS and HCQ) treatments, seven, five and four replicates were included, respectively. Also were included controls consisting of cells treated with: 50:50 PBS-culture medium (M + PBS), 1% DMSO medium, and 100% medium, in the case of NPs, α-TOS and HCQ treatments, respectively.

### 2.12. Apoptosis Detection by Acridine Orange and Ethidium bromide (AO/EtBr) Staining

Morphological changes induced by nanoparticles were evaluated using AO/EtBr dual staining under a fluorescence microscope. Briefly, cells were seeded at a density of 60,000 cells per well in a 24-well culture plate. After 24 h of incubation, the culture medium was replaced with a 1:1 (*v*/*v*) mixture of fresh medium and nanoparticles at 500 μg mL^−1^. Following 24 h and 72 h of treatment, the cells were washed with PBS and stained with equal volumes of acridine orange (AO, 1% in PBS; Fluka) and ethidium bromide (EtBr, 1 mg mL^−1^ in PBS; Sigma). Stained cells were examined immediately under a fluorescence microscope, and images were acquired for analysis. A control consisting of cells treated with 50:50 PBS-culture medium (M + PBS) was included.

### 2.13. Cell Morphology Staining

MCF-7 cells (60,000 cells per well) were seeded on coverslips placed in a 24-well culture plate to visualize cell morphology. After 24 h of incubation, the culture medium was replaced with a 1:1 (*v*/*v*) mixture of fresh medium and nanoparticles at 500 μg mL^−1^. As a control treatment, cells were exposed to 50:50 PBS-culture medium (M + PBS). Following 72 h of treatment, the NP-containing medium was removed, and the cells were washed with PBS, fixed with 3.7% paraformaldehyde (Thermo Fisher Scientific) for 15 min, and permeabilized with 0.01% Triton X-100 for 30 min at 37 °C. Subsequently, the samples were washed three times with PBS, and the cytoskeleton and nuclei were stained with Alexa Fluor^TM^ 647 Phalloidin and Hoechst (Thermo Fisher Scientific), respectively. After 3 min of incubation, the cells were washed with PBS, and images were acquired using a fluorescence microscope.

### 2.14. Statistical Analysis

A 100% viability baseline was established for a control group of untreated cells. The results for cells treated with NPs were expressed as a percentage relative to the control and recorded as mean ± standard deviation. Data sets following a normal distribution were analyzed using one-way ANOVA, followed by a least significant difference (LSD) multiple comparison test to identify differences in cell viability among the various treatment groups. In situations where cell viability did not show a normal distribution across the treatment groups (NP concentrations), a one-way rank-based Kruskal–Wallis analysis of variance (ANOVA on ranks) was performed. In these cases, differences between groups were determined by a post hoc pairwise comparison test with the Bonferroni adjustment. Statistical calculations were performed using SPSS software (version 29.0.1.0; IBM SPSS Statistics, Chicago, IL, USA) or Statgraphics Centurion XVI software (version 16.1.03, StatPoint Technologies, Inc., Warrenton, VA, USA).

## 3. Results and Discussion

### 3.1. Synthesis and Characterization of the HCQM Monomer via ^1^H-NMR

The HCQM monomer was successfully synthesized and purified, yielding 8.6 g of a viscous liquid with a greenish color and a reaction yield of 88%. TLC plates showed a single comet-shaped spot with an Rf value of 0.53, using AcOEt:MeOH:NH_3_ (87.3:12.5:0.2) as the eluent.

**Figure 2:**
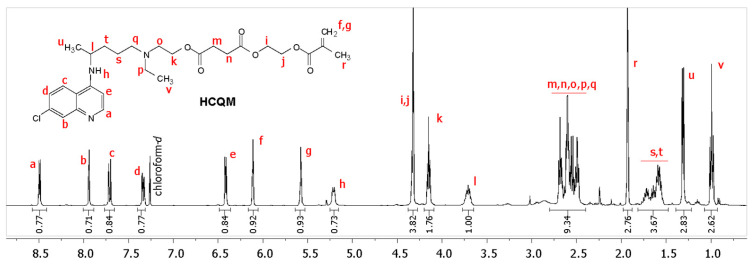
^1^H NMR spectrum of HCQM in CDCl_3_.

The ^1^H NMR spectrum of the HCQM monomer, shown in Figure 2, confirms the compound’s purity with no evidence of contamination. The vinyl protons of the methacryloyloxy group, identified as **f** and **g**, appear as well-defined singlets at 6.11 ppm (1H) and 5.58 ppm (1H), respectively. The signals in the range of 6.41 to 8.48 ppm were attributed to the aromatic protons of the quinoline ring: **a**, **b**, **c**, **d**, and **e**. Proton **d** at δ 7.34 ppm (dd, J = 8.9, 1.8 Hz, 1H) exhibits ortho coupling with proton **c** at δ 7.71 ppm (d, J = 9.0 Hz, 1H) and meta coupling with proton **b** at δ 7.94 ppm (d, J = 1.7 Hz, 1H), suggesting the presence of a chlorine substituent between b and d on the benzenoid portion of the quinoline ring. The signals at δ 6.41 ppm (d, J = 5.5 Hz, 1H) and δ 8.48 ppm (t, J = 6.1 Hz, 1H) were assigned to the aromatic protons **e** and **a** of the pyridinic portion of the quinoline system. A broad signal at δ 5.21 ppm (d, J = 7.1 Hz, 1H) was attributed to proton **h** of the cyclic amino –NH group. The signals at δ 1.28 ppm (m, 3H) and δ 3.71 ppm (dt, 1H) correspond to the aliphatic protons **u** and **l** of the CH_3_-CH- group, respectively, while the methylene protons **s** and **t** appear as multiplets at δ 1.81–1.47 ppm (4H). The methyl group of the –N–CH_2_–CH_3_ moiety appears as a signal at δ 0.99 ppm (t, J = 7.1 Hz, 3H). The set of signals at δ 2.80–2.40 ppm (m, 10H) originates from a combination of sources, including the three methylene groups directly attached to the tertiary amine (**o**, **p**, **q**) and the two methylene groups of the succinyl moiety (**m** and **n**). The triplet observed at δ 4.20–4.09 ppm (t, 2H) is attributed to the oxymethylene protons **k** of the ester group formed between MES and HCQ. The singlet at δ 4.33 ppm (s, 4H) corresponds to the **i**–**j** protons of the ethylenedioxy group, while the methyl protons **r** of the methacrylate group appear as a singlet at δ 1.93 ppm (s, 3H).

### 3.2. Synthesis and Characterization of the HCQM-VP Copolymers via ^1^H-NMR

**Figure 3:**
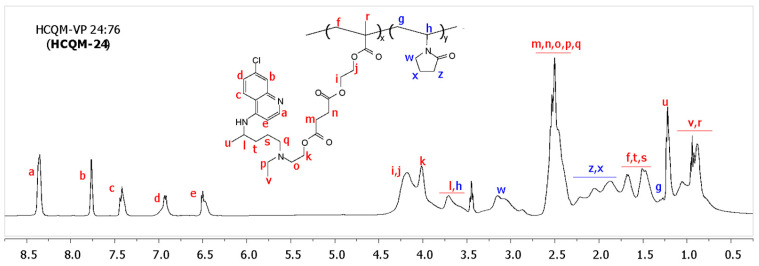
^1^H NMR spectrum of poly(HCQM-*co*-VP) 24:76 in DMSO-*d*_6_.

The HCQM-VP copolymers were successfully synthesized, resulting in a whitish powder with yields detailed in Table 1. The ^1^H-NMR spectra of the copolymers exhibit similar characteristics, with variations in signal intensities reflecting the different comonomer molar compositions. Figure 3 presents, as an example, the ^1^H-NMR spectrum of the HCQM-24 copolymer, where residual monomer signals are absent, as observed in the spectra of the other copolymers, confirming successful purification. In the mentioned spectrum, the range from 0.5 to 4.5 ppm shows an overlap of signals corresponding to both monomer residues.

In this region, the signals generated by the HCQM monomer unit correspond to the following groups: oxymethylene (**k**), succinyl (**m**, **n**), ethylenedioxy (**i**, **j**), methine (**l**), methyl (**r**, **v**, **u**), and methylenes attached to the N atom of the tertiary amine (**o**, **p**, **q**), those adjacent to these (**t**, **s**), and the main-chain one (**f**). On the other hand, the signals **h**, **w**, **z**, **x**, and **g** were assigned to protons of the VP monomer unit, including those from the methine and methylene groups attached to the nitrogen of the pyrrolidone ring (**h** and **w**, respectively), more shielded CH_2_ groups from this ring (**x**, **z**), and methylene present in the polymer backbone (**g**). Additionally, in the region between 6.0 and 8.5 ppm, the signals **a**, **b**, **c**, **d**, and **e** corresponding to the aromatic protons of the quinoline ring are observed.

The molar fractions of the HCQM and VP monomers in the copolymers were determined from the integration values of the NMR signals specific to each monomer. For HCQM, the signal set between 6.3 and 8.5 ppm was used, while for VP, the range between 0.2 and 4.7 ppm was employed. These integration values were obtained using the MestReNova software (version 6.0.2). The calculation of the molar fractions was performed using the following system of equations:

(2) $$H=\frac{\left(I_{6.3-8.5\;ppm}\right)}{5}$$

(3) $$V=\frac{\left(I_{0.2-4.7\;ppm}\right)-32H}{9}$$

(4) $$f_{HCQM}=\frac{H}{V+H}$$

(5) $$f_{VP}=\frac{V}{V+H}$$

In these equations, *H* represents the contribution of a proton from HCQM to the integration area of the signals between 6.3 and 8.5 ppm (*I*_6.3–8.5 ppm_), and *V* represents the contribution of a proton from VP to the integration area of the signals between 0.2 and 4.7 ppm (*I*_0.2–4.7 ppm_). From the values of *H* and *V*, the molar fractions of HCQM (*f*_HCQM_) and VP (*f*_VP_) in the copolymer were calculated. Table 1 shows the molar fractions of HCQM (*f*_HCQM_) for each of the synthesized copolymers.

### 3.3. DSC Analysis of HCQM-VP Copolymers and Homopolymers

The glass transition temperature (T_g_) of each synthesized polymer was recorded in Table 1. It was not possible to measure the T_g_ for the homopolymer PHCQM and the HCQM-VP copolymer with an 80:20 feeding composition due to its highly sticky consistency, which made them difficult to handle. Additionally, these samples were insoluble in all tested organic solvents (DMF, DMSO, THF, chlorinated solvents, etc.), which suggests possible crosslinking in the polymer chains.

**Figure 4:**
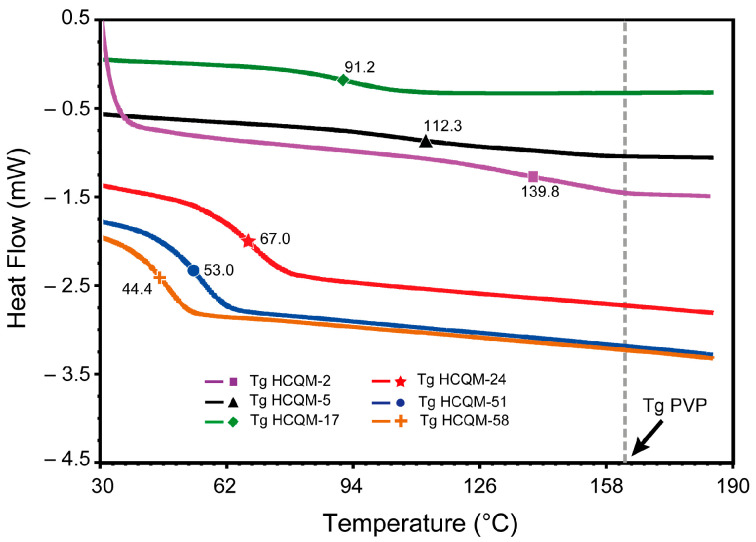
DSC curves of poly(HCQM-*co*-VP) copolymers.

Figure 4 shows a single T_g_ in the DSC curves of the copolymers, confirming the formation of random-type copolymers and the absence of homopolymer mixtures, as no microphase separation is observed. The T_g_ values of the copolymers were lower than the T_g_ of the PVP homopolymer (163.4 °C) and showed a decreasing trend with the increase in the molar fraction of the HCQM monomer (from HCQM-2 to HCQM-58), suggesting that the HCQM units act as a plasticizer, increasing the mobility of the polymer chains [25]. This behavior can be attributed to the greater flexibility and steric volume of the HCQM units compared to those of VP, which decreases the packing density and weakens the strong dipole–dipole intra- and inter-chain attractions between the VP units [26].

The flexibility of the HCQM units is mainly due to the long-chain aliphatic spacer that separates the quinoline ring from the polymer backbone [27]. In contrast, the VP unit is a cyclic amide directly attached to the polymer chain, which experiences dipolar attractive interactions with other VP units. It therefore has fewer degrees of freedom for movement. As a result, an increase in the molar fraction of VP generates greater attraction and cohesion between the chains, reducing the free volume and leading to an increase in T_g_ [28,29].

Moreover, the increase in T_g_ for higher VP compositions would also be associated with increasing segregation (less uniformity in the distribution) of comonomers along the polymer chain. This implies that one chain end becomes richer in VP units and the other in HCQM units as the VP molar fraction increases in the copolymers. On the other hand, the decreasing trend in T_g_ with increasing HCQM compositions suggests greater uniformity in the comonomer distribution within the copolymers [30]. This possible distribution is inferred based on the results of several studies that demonstrate a strongly non-ideal behavior of VP when copolymerized with methacrylic monomers, due to reactivity ratios close to zero (0) for the former and greater than one (>1) for the latter [31,32,33,34].

### 3.4. GPC Analysis of HCQM-VP Copolymers and Homopolymers

Table 1 shows the weight-average molar mass (M_w_) and molar mass dispersity (Ð = M_w_/M_n_) values of the HCQM-VP copolymers determined using the GPC technique. Ð values ranging from 1.5 to 2.9 indicate that the growing polymer chains tend to terminate by disproportionation, which is characteristic of conventional free radical polymerization [35]. The increasing trend in Ð and M_w_ (or M_n_) values with the molar fraction of HCQM in the copolymer (*f*_HCQM_) is likely due to the increase in branching in the “dead” polymer chains [29,36]. The branching would be produced by chain transfer reactions originating from the HCQM units pendant on the main chain, which occur by abstraction of hydrogens from the ethylenedioxy group by radicals of the reaction medium, to form stabilized mid-chain radicals. These radicals can react with other monomers, primarily HCQM, to generate the branches [37,38,39,40]. The increase in branching with *f*_HCQM_ would provide an additional explanation for the observed decrease in T_g_, as branched polymers have more chain ends and, consequently, a greater amount of free volume, i.e., better segmental mobility [41,42,43].

The GPC chromatograms (Figure S1) reveal that only HCQM-2 and HCQM-5 have unimodal molar mass distributions with a broad main peak referred to as peak #1, while the rest of the copolymers exhibit a superimposed peak #1 with a second peak slightly shifted to the left, referred to as peak #2. Additionally, only in HCQM-17 and HCQM-24 is there a relatively well-resolved peak #3, of very low intensity and located at lower elution times (higher molar masses), which should not be confused with peak #2 in HCQM-17, as in this case, peak #2 appears more like a remnant shoulder of peak #1. Note that the height of peak #2 relative to peak #1 gradually increases with the molar fraction of HCQM in the copolymer, reaching a maximum for HCQM-58, which is approximately 70% higher than that of peak #1.

Each of the three types of peaks (#1, #2, and #3) in the chromatograms could be associated with a different type of polymer architecture, which might be present among the macromolecules of the respective copolymers. In this regard, peak #1 could correspond to a majority fraction of linear polymer chains. Peak #2 is attributed to the branched chains described above, while peak #3 could correspond to a minimal fraction of polymer chains with longer and more numerous branches than those associated with peak #2. Finally, the insolubility of the two polymers with the highest molar fraction of HCQM in the feed, i.e., polyHCQM and HCQM-VP 80:20, can be explained by the high density of crosslinking points achieved between their linear polymer chains, which eventually formed an infinite network structure. This network is generated when the previously mentioned half-chain radicals react with growing polymer chains, forming long connections between linear polymers (crosslinking points) [37,44].

### 3.5. Thermogravimetric Analysis of HCQM-VP Copolymers

Table 2 and Figure S2 (see Supplementary Materials) show the results of the thermogravimetric analysis (TGA) of PVP and the HCQM–VP copolymers. The DTG thermogram of the PVP homopolymer revealed a single decomposition peak at 438.7 °C, attributed to the depolymerization of the polymer’s main chain. Although it was not possible to analyze the thermal degradation profile of the PHCQM homopolymer, the results obtained for PVP provide a key reference for understanding the thermal behavior of the copolymers.

In contrast to the behavior of PVP, the TGA thermograms of the HCQM–VP copolymers exhibit two well-defined decomposition stages. The first stage occurs between 225 and 350 °C and involves a weight loss of 11–44%, possibly associated with the cleavage of ester bonds in the spacer (MES-derived fragment) that links the HCQ molecule to the main chain of the copolymer. As the HCQM content in the copolymers increases, the temperature corresponding to this stage (TgA1) remains relatively constant at around 304–308 °C, while the weight loss (ΔW1) progressively increases from 10.8% for HCQM-2 to 44.0% for HCQM-58. This behavior suggests that in the first stage, the degradation of the HCQM monomeric units in the copolymer is likely taking place.

The second stage of degradation, occurring between 350 and 500 °C with peaks ranging from 432 to 439 °C, involves a weight loss of 41–80% (80.1% in HCQM-2 to 41.2% in HCQM-58) and corresponds to the weight of the polymer fraction that did not degrade in the first stage, that is: the MES spacer residues (without the HCQ molecule) together with the depolymerization and fragmentation of the polymer main chain. The previously described thermal degradation processes confirm the successful copolymerization of HCQM and VP.

### 3.6. Characterization of α-TOS-Loaded and Empty Poly(HCQM-co-VP) Nanoparticles

The copolymers HCQM-5, -17, and -24 displayed an appropriate hydrophobic/hydrophilic balance, allowing the formation of nanoparticles in PBS upon nanoprecipitation using THF:DMF (7:3) as the solvent. However, when this solvent was replaced with the THF:DMF:DMSO mixture (86:7:7), only HCQM-17 and -24 successfully formed nanoparticles. The remaining copolymers (HCQM-2, -51, and -58), regardless of the solvent used, formed aggregates upon contact with PBS and adhered to the glass walls of the vial, preventing their evaluation by DLS. The two organic solvent systems were tested to modulate the particle size and adjust it to the 50–150 nm range, which is considered optimal for use in antitumor drug delivery systems [45].

In Table 3, the hydrodynamic diameter (Dh) and polydispersity index (PDI) of the nanoparticles obtained with the copolymers and solvents mentioned above are presented. The nanoparticles are hereafter designated with the abbreviation “NP” followed by the name of the corresponding copolymer (according to Table 1) for those prepared with the THF:DMF (7:3) solvent. The designation is complemented by the letter “A” (at the end) if the nanoparticles were prepared with the THF:DMF:DMSO (86:7:7) solvent mixture. Note that among the empty NPs, only NP-HCQM-17 showed Dh (123.6 nm) and PDI (0.103) values within the range suggested to experience the EPR effect, i.e., 50 < Dh < 150 nm and PDI < 0.3 [45]. This size range was exceeded by NP-HCQM-5, NP-HCQM-17-A, NP-HCQM-24-A, and NP-HCQM-24, and therefore, they were discarded for α-TOS encapsulation. It is important to highlight that NP-HCQM-17 did not present visible precipitates for at least 20 days at room temperature.

**Figure 5:**
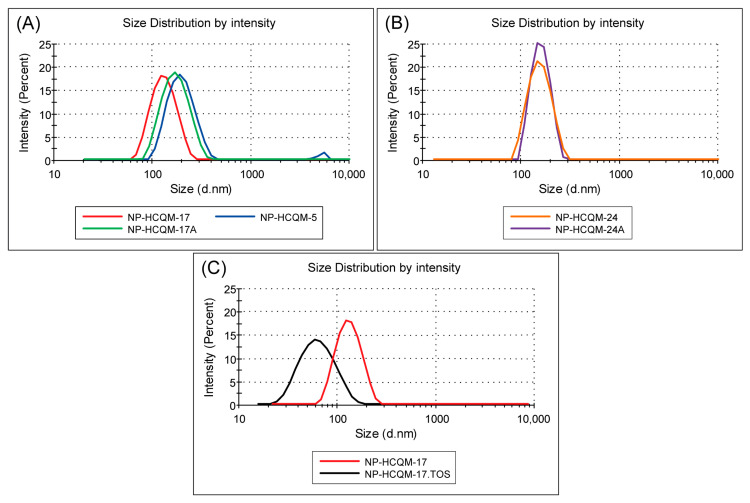
DLS size distribution curves of: (**A**) NP-HCQM-17, -17A and -5; (**B**) NP-HCQM-24 and -24A, and (**C**) empty NP-HCQM-17 versus α-TOS loaded NP-HCQM-17.

When comparing the DLS curves of the five types of empty nanoparticles (Figure 5A,B), it is evident that all size distributions were unimodal, indicating uniform hydrodynamic diameter, except for NP-HCQM-5, which was bimodal (with the highest PDI value, 0.24), and therefore more heterogeneous.

**Figure 6:**
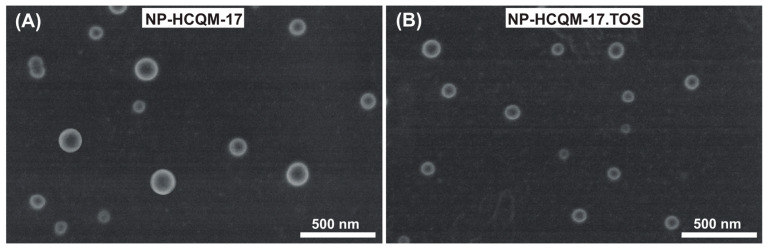
SEM electron micrograph of empty (**A**) and α-TOS-loaded (**B**) NP-HCQM-17.

The nanoparticles in the present study likely adopted a core–shell micellar structure, with amphiphilic polymer chains enriched at one end with VP units (hydrophilic), oriented towards the external surface of the micelle (shell), and the other end enriched with HCQM units (hydrophobic), oriented towards the core. SEM images of NP-HCQM-17 confirmed a spherical morphology (Figure 6). The concept of an amphiphilic polymer that can form micelles is based on previous studies that attribute reactivity ratios greater than one and close to zero for methacrylates (e.g., HCQM) and VP, respectively [12], as explained in the previous section on the DSC results of the copolymers.

Table 3 shows negative zeta potential values for the NPs, which can be explained as follows: the hydrophilic VP units of the polymer chains, mostly distributed on the external surface of the NPs (crown), form a Stern layer with a positive charge due to Na^+^ and K^+^ cations (from PBS) attracted to the lactam carbonyl group, which in turn attract anions (especially chloride) to the diffuse layer, resulting in a negative zeta potential [11].

The comparison between the NP-HCQM-17 system and its version prepared with the solvent containing DMSO (i.e., NP-HCQM-17A) shows that the latter has a higher Dh value (167.1 vs. 123.6 nm) and a more negative zeta potential (−18.5 vs. −5.8 mV) (see Table 3, Figure 5A). According to previous studies, this behavior could be attributed to the high viscosity and elevated dielectric constant of the DMSO solvent, which slows down the nucleation and aggregation rate during the self-assembly of the polymer chains, leading to the formation of cores with a higher number of polymer chains per micelle. This, along with the ability of DMSO to redistribute surface charges, not only increases the nanoparticle size but also enhances the negative charge density on its surface, resulting in a more negative zeta potential [46,47,48,49]. On the other hand, during the nanoprecipitation of the HCQM-24 copolymer, the solvent type effect on size (Figure 5B) and zeta potential (Table 3) of the nanoparticles was less evident.

In summary, the optimal characteristics of NP-HCQM-17, such as its size of 123.6 nm, low polydispersity (PDI = 0.103), and good colloidal stability (zeta potential = −5.8 mV), led to the selection of the HCQM-17 copolymer to encapsulate the α-TOS drug, resulting in an encapsulation efficiency of 78% (78.0 ± 5.8%).

The NP-HCQM-17 loaded with α-TOS (i.e., NP-HCQM-17.TOS) exhibited a smaller diameter (Table 3) compared to its unloaded version (71.0 vs. 123.6 nm), as evidenced by both DLS (Figure 5C) and SEM (Figure 6A,B). This size reduction is likely due to α-TOS can behave as a surfactant during nanoprecipitation and reduce the number of polymer chains per micelle. Another plausible explanation would be the improved compaction of the nanoparticle core induced by hydrophobic interactions between α-TOS molecules and the HCQM-rich chain ends of the copolymer [50,51,52]. It should be noted that in the field of biomedical applications of NPs in aqueous environments, the hydrodynamic diameter determined by DLS is more relevant than that measured in dry conditions by SEM.

The high encapsulation efficiency (78%) and the increased zeta potential of the loaded nanoparticles compared to the empty ones (−19.2 vs. −5.8 mV) suggest a higher electrostatic stability of the nanoparticles due to the incorporation of α-TOS (Table 3). These results align with those from previous studies, which show that amphiphilic compounds like α-TOS can stabilize nanoparticle interfaces by orienting the polar carboxylic groups towards the surface and reducing interfacial energy [51].

Since both nanoparticles systems, NP-HCQM-17 and NP-HCQM-17.TOS, have sizes between 50 and 150 nm, they can accumulate in cancerous tumors due to the EPR effect and non-phagocytic endocytosis, without being eliminated by the reticuloendothelial mechanism. This latter mechanism easily expels nanoparticles smaller than 50 nm in diameter, while non-phagocytic endocytosis favors the internalization of those smaller than 150 nm [45]. Therefore, these nanoparticles were selected to assess their cytotoxicity in MCF-7 cell line using the AlamarBlue bioassay.

### 3.7. Cytotoxic Activity of Poly(HCQM-co-VP) Nanoparticles

**Figure 7:**
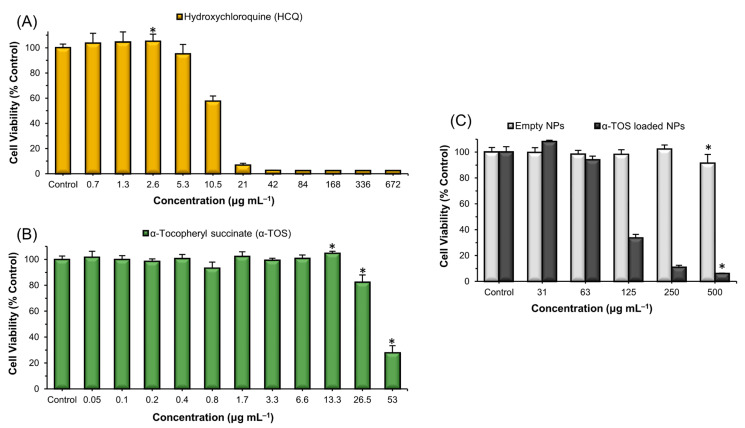
Cytotoxic effect on MCF-7 cells after 72 h of treatment with (**A**) HCQ, (**B**) α-TOS, and (**C**) different concentrations of NP-HCQM-17: empty- (grey bars) or α-TOS-loaded- (black bars). The diagrams show the mean ± standard deviation (*n* = 7, empty NP; *n* = 5, α-TOS-loaded NP; *n* = 4, α-TOS and HCQ) and the results of ANOVA or Kruskal–Wallis tests. An asterisk (∗) indicates a statistically significant difference (*p* < 0.05) compared with the control (M + PBS).

Figure 7 presents the results obtained by evaluating the cytotoxicity of the free drugs HCQ and α-TOS, as well as the nanoparticle formulations NP-HCQM-17.TOS (α-TOS-loaded) and NP-HCQM-17 (empty). These measurements were performed using the AlamarBlue assay on MCF-7 tumor cells, and the results were plotted as percentages of cell viability against the concentrations of the respective samples.

In order to compare the IC_50_ values of these 4 treatments, it was first necessary to express the concentrations of both nanoparticle formulations in HCQ and α-TOS equivalents. The amounts of α-TOS and HCQ released into the culture medium from each defined concentration of NP-HCQM-17.TOS were estimated assuming that they corresponded to the encapsulated α-TOS and copolymer-bound HCQM values, which were calculated based on %EE = 78% and *f_HCQM_* = 0.17, respectively. Consequently, the concentrations of 31, 63, 125, 250, and 500 μg mL^−1^ of NP-HCQM-17.TOS expressed in terms of α-TOS were 1.2, 2.4, 4.9, 9.8, and 19.5 μg mL^−1^, respectively, while those expressed in terms of HCQ were: 9.6, 19.2, 38.5, 77, and 154 μg mL^−1^. The concentrations of NP-HCQM-17 (empty NPs) were also expressed in terms of HCQ. The IC_50_ values of the four samples were then calculated using the AAT Bioquest IC50 Calculator online, and the results are shown in Table 4.

The NP-HCQM-17.TOS formulation was cytotoxic to MCF-7 cell after 72 h of treatment, showing an IC_50_ of 100.2 μg mL^−1^ based on the total mass of the nanosystem (Table 4). Normalizing this value to the incorporated free drugs, it is observed that the equivalent IC_50_ of α-TOS was 3.7 μg mL^−1^, representing a ninefold reduction compared to free α-TOS (IC_50_ = 32.8 μg mL^−1^). In contrast, the IC_50_ equivalent for HCQ in the same formulation (29.5 μg mL^−1^) was approximately three times higher than that of free HCQ (10.9 μg mL^−1^). These results suggest that encapsulation of α-TOS in the nanoparticles significantly enhances its own cytotoxic effect relative to the free drug. However, although covalent attachment of HCQ to the NPs appears to reduce its cytotoxic activity compared with free HCQ, its interaction with encapsulated α-TOS would promote a relatively greater availability of HCQ compared with the empty nanoparticles (NP-HCQM-17). The latter formulation showed a very high IC_50_ (>154 μg mL^−1^), suggesting negligible inhibitory activity, which could be attributed to a higher resistance to hydrolytic agents compared with the α-TOS-loaded counterpart (Table 4, Figure 7C). The high stability of the polymeric micelles in the absence of α-TOS is likely due to strong intra- and interchain attractive interactions via π-π stacking between the HCQM units of the copolymeric chains, which would limit the release of the HCQ [11,53,54].

On the other hand, the marked cytotoxicity of α-TOS-loaded nanoparticles compared to empty NPs could be attributed both to their smaller size (71.0 vs. 123.6 nm) and to their lower micellar stability. The latter could be explained by assuming that α-TOS: (1) weakens the attractive interactions between the chains ends of the core (due to incompatible molecular topologies), and (2) is distributed heterogeneously across the core and core–corona interface promoting aqueous channel formation that would facilitate nanoparticle hydrolytic or enzymatic degradation and, consequently, greater drug release (α-TOS and HCQ) compared with to empty NPs. The hydrolysis of the HCQM side groups of the copolymer would disrupt its hydrophobic/hydrophilic balance, thereby leading to micelle disintegration. Consequently, α-TOS would also be released into the intracellular environment along with HCQ, amplifying the cytotoxic effect in MCF-7 cancer cells line treated with NP-HCQM-17.TOS. However, HCQ release remains incomplete, which explains why nanoparticles’ effect (IC_50_) does not reach the levels of the free drug.

### 3.8. Apoptosis Detection by (AO/EtBr) Staining

**Figure 8:**
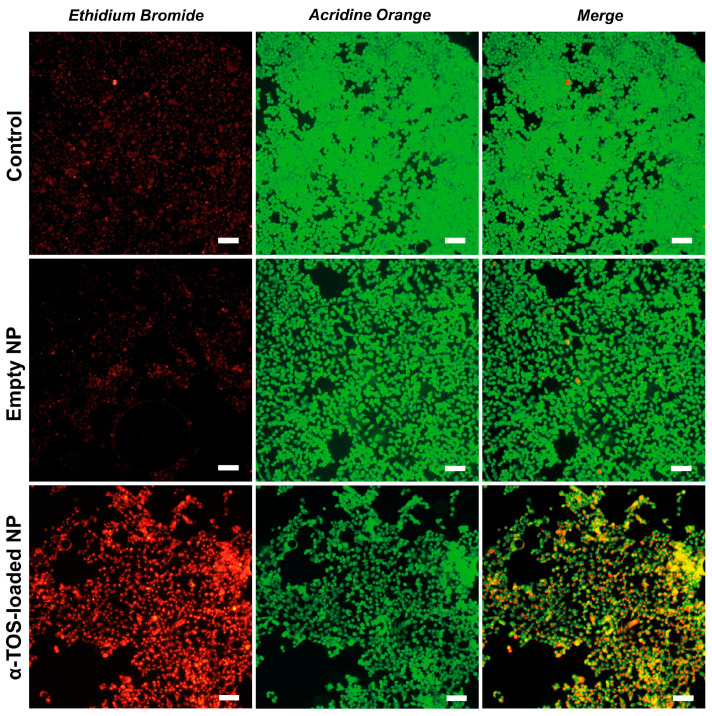
Dual acridine orange/ethidium bromide (AO/EtBr) staining of MCF-7 cells after treatment with the control (M + PBS), empty and α-TOS-loaded NP-HCQM-17 for 72 h. Live cells are uniformly green. Early apoptotic cells are stained green to yellow and contain dots in their nuclei (chromatin fragmented). Late apoptotic cells have orange chromatin that is highly condensed or fragmented. Necrotic cells are shown as deep red. A scale bar of 100 μm was used.

Double staining with acridine orange (AO) and ethidium bromide (EtBr) allowed the assessment of cell viability and the death type through morphological changes in the nucleus in MCF-7 cell after treatment with empty and α-TOS-loaded NP-HCQM-17 for 24 (Figure S3) and 72 h (Figure 8). AO stains both live and apoptotic cell due to its ability to cross intact cell membranes, whereas EtBr only penetrates cells with lost membrane integrity. In this way, it is possible to differentiate four different classes of nuclei: (i) normal cells (bright green chromatin with an organized structure), (ii) early apoptotic cells (highly condensed or fragmented bright green chromatin), (iii) late apoptotic cells (highly condensed or fragmented bright orange chromatin), and (iv) necrotic cells (intense red staining) [55].

The results obtained showed that, at both incubation times, the control cells showed a higher intensity of green fluorescence, with uniform distribution and normal morphology, without evidence of nuclear condensation or loss of membrane integrity, reflecting the high viability of the control culture.

Similarly, treatment with empty nanoparticles did not induce significant changes in cell morphology or viability compared with the control. Cell integrity was maintained, suggesting that these nanoparticles do not induce significant cytotoxicity. Most cells remained green, with organized chromatin and no evidence of nuclear fragmentation, although slight decreases in density were observed, reflecting loss of contact between neighboring cells. Only a few isolated EtBr-stained, nuclei were observed, indicating that empty nanoparticles do not induce appreciable cell death.

In contrast, cells treated with loaded nanoparticles showed an increasing proportion of yellow- and orange-stained nuclei, reflecting early and late apoptosis, respectively. At 24 h, characteristic morphological changes in apoptosis such as membrane blebbing, nuclear condensation, and fragmentation were observed, along with fluorescent yellow (early apoptosis) and orange (late apoptosis) nuclei (Figure S3, Supplementary Materials) [56]. At 72 h, the apoptotic effect was more pronounced, with green fluorescence markedly decreasing compared to the control, while the red EtBr signal increased, indicating progressive loss of membrane integrity. Overlaying both stainings showed a large number of fluorescent orange nuclei, confirming advanced apoptosis, in addition some red cells corresponding to secondary necrosis (Figure 8).

Taken together, these results allow us to conclude that α-TOS-loaded NP-HCQM-17 effectively induce apoptotic cell death in MCF-7 cancer cells, while empty NP-HCQM-17 do not alter cell viability. Similarly, previous studies have reported that α-TOS induces apoptosis in MCF-7 cells by mitochondrial mechanisms. Furthermore, NPs based on amphiphilic copolymers encapsulating α-TOS have been shown to induce intrinsic apoptotic cell death via complex II of the mitochondrial electron transport chain [51].

### 3.9. Cell Morphology

**Figure 9:**
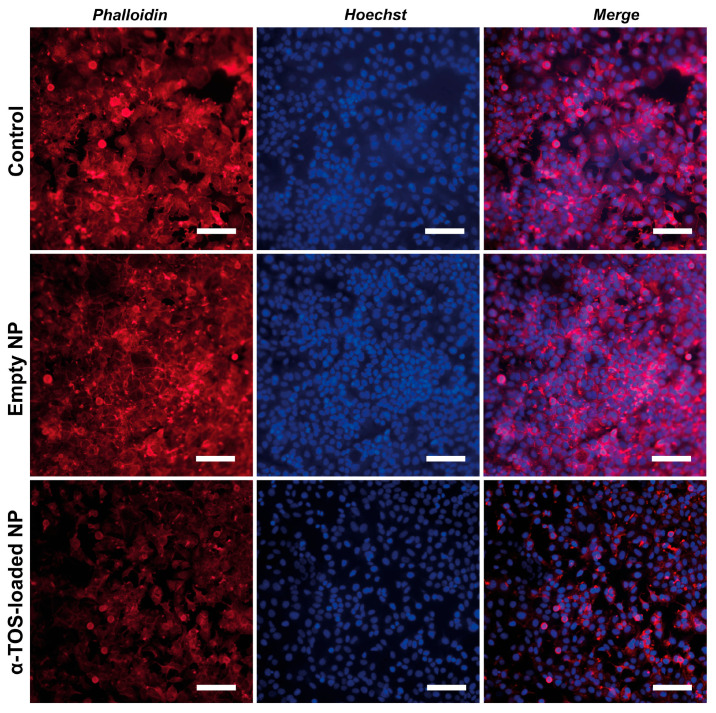
Fluorescence microscopy images of MCF-7 cells after 72 h of treatment with the control (M + PBS), empty NP-HCQM-17, and α-TOS-loaded NP-HCQM-17. Red staining indicates the cytoplasm and blue staining indicates the nucleus. The merged images show the combined staining of cytoplasm and nucleus. A scale bar of 100 μm was used.

Cytoplasmic and nuclear staining provides a general overview of the morphology state of cell after 72 h of treatment (Figure 9).

In the control group and in cells treated with empty nanoparticles, a well-defined cytoplasm and uniform nuclei were maintained, with no evidence of significant morphological alterations. Furthermore, the cell density observed in the treatment with empty nanoparticle is similar to that of the control, suggesting that they do not exert a relevant cytotoxic effect. In contrast, cells treated with α-TOS-loaded nanoparticles showed a significant reduction in cell density and loss of the ability to form colonies, indicating that the treatment induces cell death. When relating these findings with those obtained by double staining with AO/EtBr, it is confirmed that the reduction in colony formation is directly associated with the progressive apoptotic effect induced by the loaded NPs. These results are particularly relevant considering that rapid and uncontrolled and rapid proliferation is a distinctive feature of malignant tumors [57].

According to optical microscopy analyses of MCF-7 cultures, a qualitative reduction in cell number and significant morphological changes were observed in the presence of both empty and α-TOS-loaded NP-HCQM-17 compared with the control group. These changes were especially pronounced in the case of NP-HCQM-17.TOS, where cell shrinkage and the formation of apoptotic bodies were observed (Figures S4 and S5, Supplementary Materials). These morphological results support the in vitro cytotoxicity results.

## 4. Conclusions

A series of copolymers were successfully synthesized from a hydroxychloroquine-derived monomer (HCQM) and N-vinylpyrrolidone (VP) via conventional radical polymerization. The copolymer HCQM-17 achieved a hydrophobic/hydrophilic balance that allowed it to self-assemble in an aqueous medium, forming nanoparticles with a hydrodynamic diameter of 123.6 nm and stability suitable for its application in cancer treatment. These nanoparticles behaved as excellent nanocarriers, effectively encapsulating α-TOS in their hydrophobic core, achieving an encapsulation efficiency (%EE) of 78%, with a smaller diameter and more negative zeta potential compared to the empty NPs.

In vitro evaluation of their cytotoxic activity using the AlamarBlue assay on MCF-7 cell line showed that the empty NPs were innocuous, while the α-TOS-loaded NPs exhibited significant cytotoxicity (IC_50_ = 100.2 μg mL^−1^). AO/EtBr staining showed apoptosis induced by loaded nanoparticles, while empty nanoparticles did not affect cell viability. In addition, the colony formation assay confirmed the loss of cell proliferation as a consequence of this mechanism. These results highlight the potential of NPs-HCQM-17 as a controlled-release system for α-TOS (or other hydrophobic drugs) for the treatment of cancer (or other diseases treatable with these drugs), contributing to the development of HCQ-based polymeric nanocarriers.

## Figures and Tables

**Table 1:** Feed (F) and copolymer (*f*) HCQM molar fractions, reaction yields, glass transition temperature (T_g_), molecular weights (M_w_ and M_n_) and polydispersity indices (M_w_/M_n_ = Ð) for the synthesized poly(HCQM-*co*-VP).

Copolymer	F_HCQM_	*f*_HCQM_	Yield (%)	T_g_ (°C)	M_w_ (kDa)	M_n_ (kDa)	Ð
HCQM-2	0.05	0.02	58.9	139.8	11.6	7.7	1.5
HCQM-5	0.10	0.05	45.7	112.3	11.7	7.5	1.6
HCQM-17	0.20	0.17	51.1	91.2	26.6	9.2	2.9
HCQM-24	0.30	0.24	52.4	67.0	20.8	10.2	2.0
HCQM-51	0.50	0.51	52.9	53.0	26.0	14.4	1.8
HCQM-58	0.70	0.58	43.1	44.4	33.9	15.6	2.2

**Table 2:** Results of thermogravimetric analysis (TGA) of the synthesized PVP and the poly(HCQM-*co*-VP).

Polymer	TgA1 (°C)	ΔW1	TgA2 (°C)	ΔW2
PVP	---	---	438.7	89.8
HCQM-2	306.0	10.8	436.5	80.1
HCQM-5	304.8	17.8	434.2	74.3
HCQM-17	308.6	31.4	432.4	57.8
HCQM-24	306.5	36.2	435.7	51.7
HCQM-51	307.6	42.5	434.0	42.8
HCQM-58	304.5	44.0	438.0	41.2

**Table 3:** Hydrodynamic diameter (Dh, by intensity), polydispersity index (PDI), zeta potential (ZP) and α-TOS weight percentage of poly(HCQM-*co*-VP) NPs of different copolymer HCQM molar fractions (*f*_HCQM_) prepared at 1000 μg mL^−1^ in PBS by nanoprecipitation. Data were obtained by DLS (Zetasizer Lab, Malvern).

Sample	*f*_HCQM_	Dh (nm)	PDI	ZP (mV)	α-TOS (%)	EE (%)	* Solvent
NP-HCQM-5	0.05	180.9 ± 5.4	0.240 ± 0.034	−17.8 ± 4.5	0	---	T + D
NP-HCQM-17A	0.17	167.1 ± 2.2	0.090 ± 0.042	−18.5 ± 3.2	0	---	T + D + DMSO
NP-HCQM-17	0.17	123.6 ± 3.5	0.103 ± 0.015	−5.8 ± 4.2	0	---	T + D
NP-HCQM-17.TOS	0.17	71.00 ± 2.8	0.168 ± 0.028	−19.2 ± 2.5	10	78.0 ± 5.8	T + D
NP-HCQM-24A	0.24	162.9 ± 2.4	0.160 ± 0.048	−23.8 ± 5.2	0	---	T + D + DMSO
NP-HCQM-24	0.24	152.9 ± 3.2	0.024 ± 0.020	−24.5 ± 2.8	0	---	T + D

*: T + D = THF:DMF (70:30); T + D + DMSO = THF:DMF:DMSO (86:7:7).

**Table 4:** IC_50_ Values for MCF-7 Cell Line Incubated with the Samples for 72 h.

Sample	IC_50_ NP (μg mL^−1^)	IC_50_ Equiv. α-TOS (μg mL^−1^)	IC_50_ Equiv. HCQ (μg mL^−1^)
NP-HCQM-17.TOS	100.2	3.7	29.5
α-TOS	---	32.8	---
HCQ	---	---	10.9
NP-HCQM-17	>500	---	>154

## Data Availability

The original contributions presented in this study are included in the article/Supplementary Material. Further inquiries can be directed to the corresponding author.

## Supplementary Materials

The following supporting information can be downloaded at: https://www.mdpi.com/article/10.3390/polym17192672/s1, Figure S1: GPC chromatograms of the synthesized poly (HCQM-*co*-VP) copolymers; Figure S2: Thermogravimetric analysis (TGA) curves of poly(HCQM-*co*-VP) copolymers and PVP homopolymer; Figure S3: Dual acridine orange/ethidium bromide (AO/EtBr) staining of MCF-7 cells after treatment with the control (M+PBS), empty and α-TOS-loaded NP-HCQM-17 for 24 h. A scale bar of 100 μm was used; Figure S4: Representative optical micrographs of MCF-7 cells after 24h (top panel) and 72 h (bottom panel) of treatment with 53 μg mL^−1^ α-TOS. Scale bar 100 μm; Figure S5: Representative optical micrographs of MCF-7 cells after 24h (top panel) and 72 h (bottom panel) of treatment with differents concentratios of HCQ. Scale bar 100 μm.
