# Supplementary material for: Polymeric Nanovehicle of α-Tocopheryl Succinate Based on a Methacrylic Derivative of Hydroxychloroquine and Its Cytotoxic Effect on Breast Cancer Cells
Source: Polymers (Basel). 2025 Oct 2;17(19):2672. doi: 10.3390/polym17192672 (PMC12526677; doi:10.3390/polym17192672)
Supplement: Supplementary file 1 [file polymers-17-02672-s001.zip › polymers-3701912-supplementary.pdf]

polymers

MDPI

Supporting material

Polymeric Nanovehicle of α-Tocopheryl Succinate Based on a Methacrylic Derivative of Hydroxychloroquine and Its Cytotoxic Effect on Breast Cancer Cells

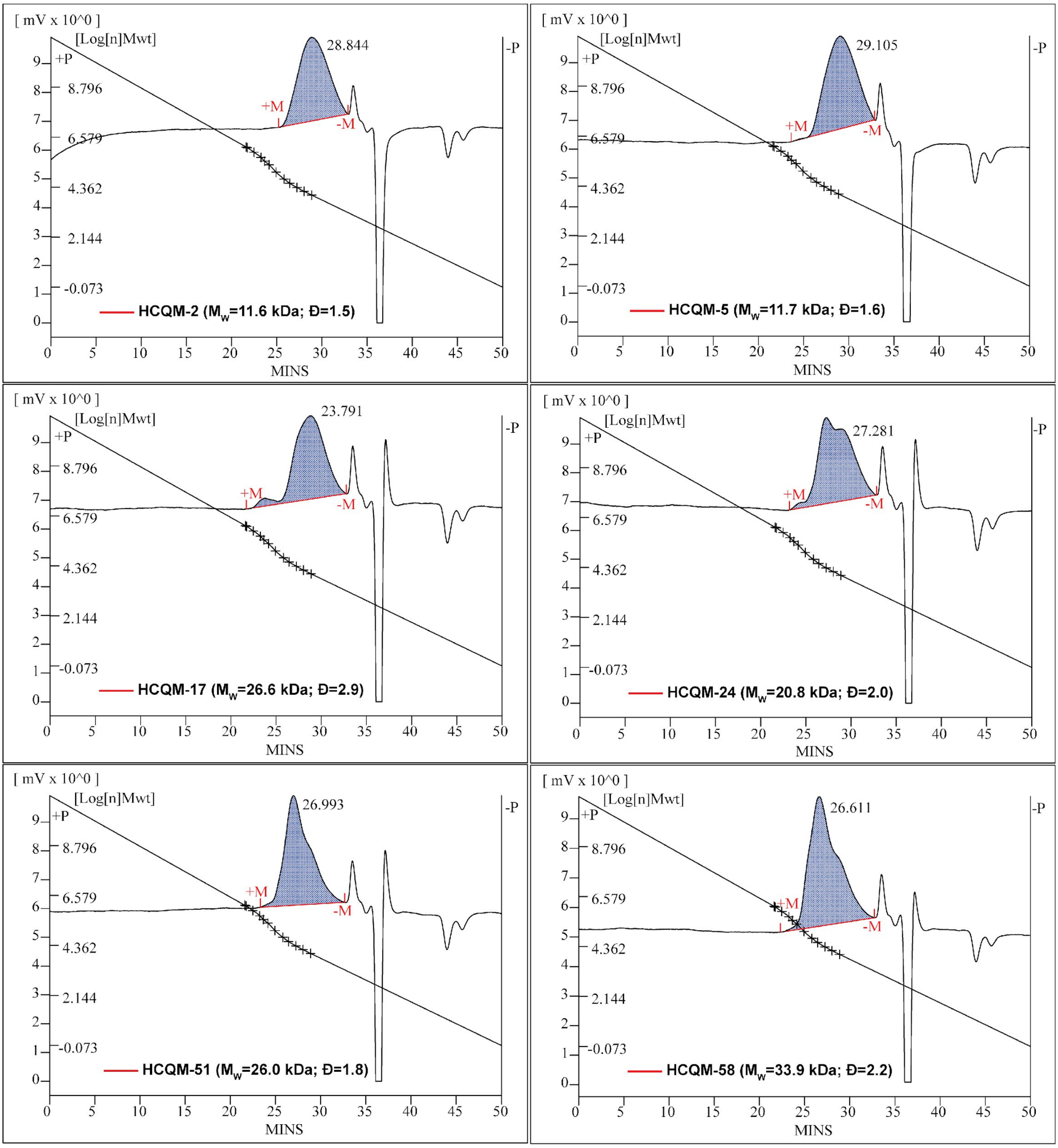

Figure S1. GPC chromatograms of the synthesized poly(HCQM-*co*-VP) copolymers.

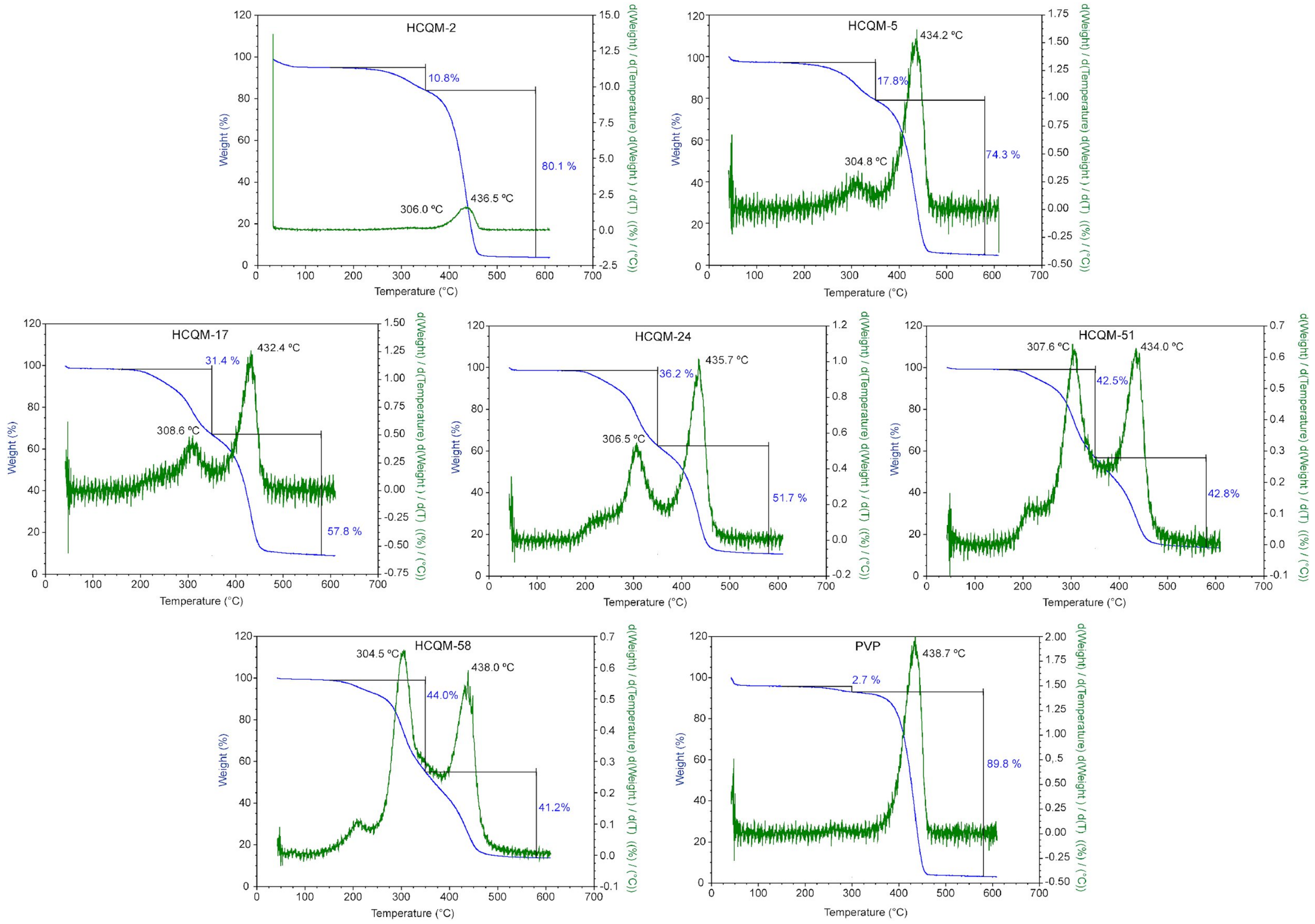

Figure S2. Thermogravimetric analysis (TGA) curves of poly(HCQM-*co*-VP) copolymers and PVP homopolymer.

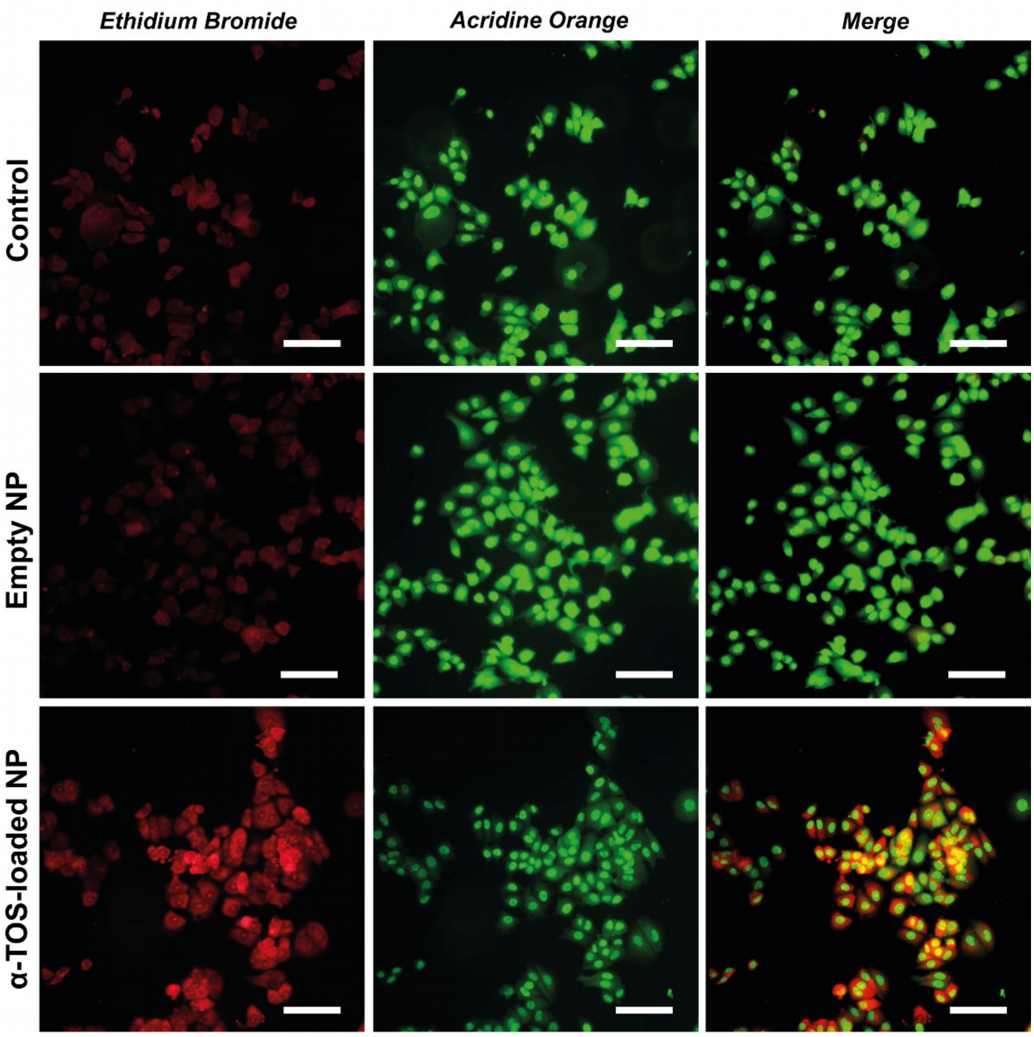

Figure S3. Dual acridine orange/ethidium bromide (AO/EtBr) staining of MCF-7 cells after treatment with the control (M+PBS), empty and a-TOS-loaded NP-HCQM-17 for 24 h. A scale bar of 100 mm was used.

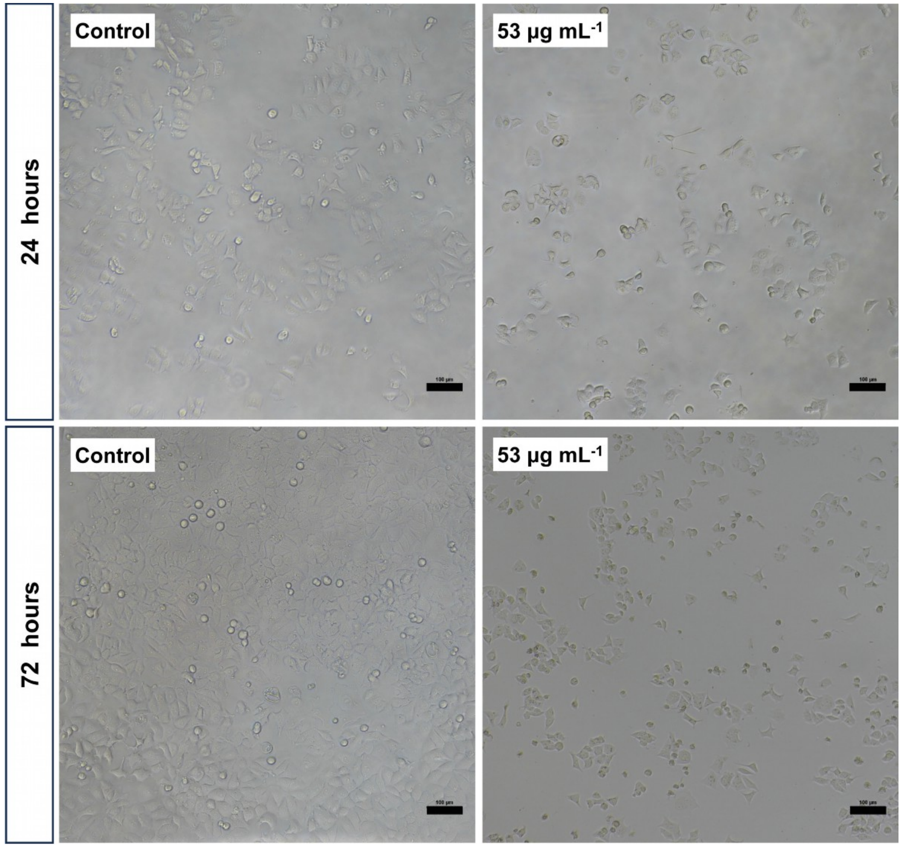

Figure S4. Representative optical micrographs of MCF-7 cells after 24h (top panel) and 72 h (bottom panel) of treatment with 53 µg mL^{-1} a-TOS. Scale bar 100 µm.

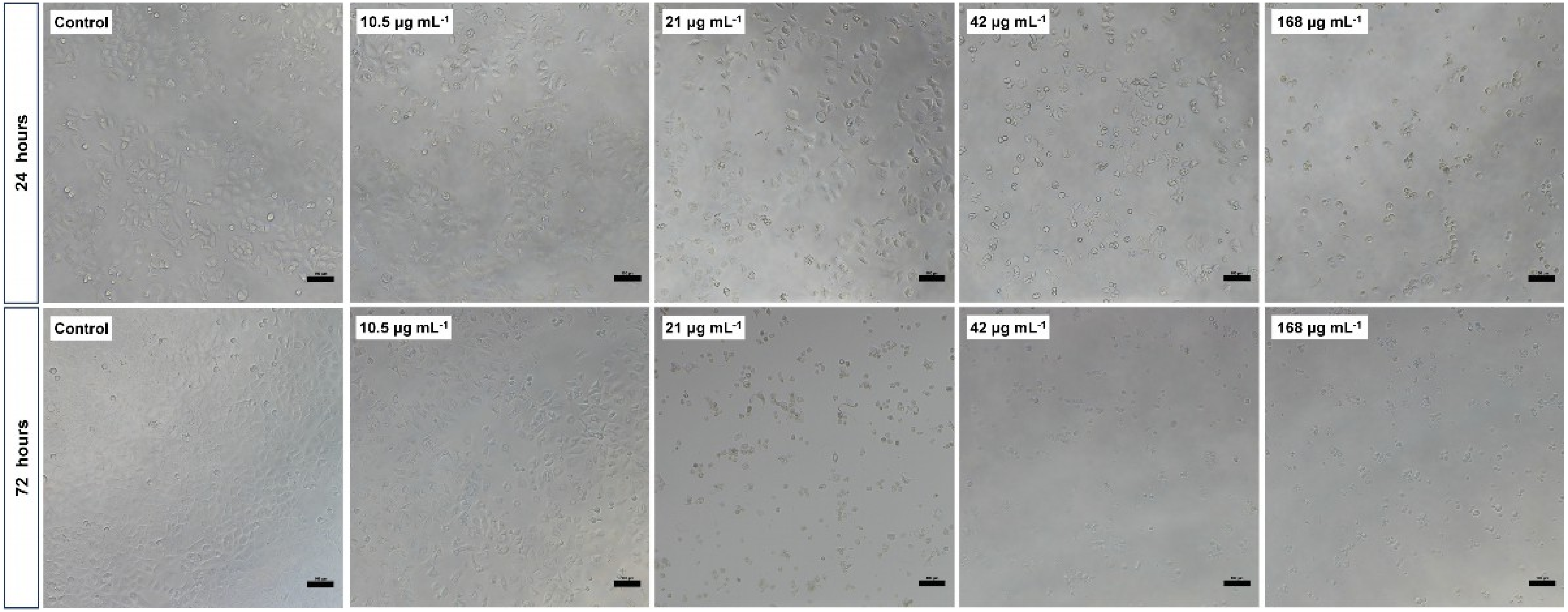

Figure S5. Representative optical micrographs of MCF-7 cells after 24h (top panel) and 72 h (bottom panel) of treatment with differents concentratios of HCQ. Scale bar 100 µm.
